# Unlocking bat immunology: establishment of *Pteropus alecto* bone marrow-derived dendritic cells and macrophages

**DOI:** 10.1038/srep38597

**Published:** 2016-12-09

**Authors:** Peng Zhou, Yok Teng Chionh, Sergio Erdal Irac, Matae Ahn, Justin Han Jia Ng, Even Fossum, Bjarne Bogen, Florent Ginhoux, Aaron T Irving, Charles-Antoine Dutertre, Lin-Fa Wang

**Affiliations:** 1 Programme in Emerging Infectious Disease, Duke-NUS Medical School, 8 College Road, 169857 Singapore, Singapore; 2 K.G. Jebsen Center for Research on Influenza Vaccines, Oslo University Hospital, University of Oslo, 0027 Oslo, Norway; 3 Center for Immune Regulation, Institute of Immunology, Oslo University Hospital Rikshospitalet, University of Oslo, 0424 Oslo, Norway; 4 Singapore Immunology Network (SIgN), Agency for Science, Technology and Research (A*STAR), 8A Biomedical Grove, IMMUNOS Building #3-4, BIOPOLIS, 138648 Singapore, Singapore

## Abstract

Bats carry and shed many emerging infectious disease agents including Ebola virus and SARS-like Coronaviruses, yet they rarely display clinical symptoms of infection. Bat epithelial or fibroblast cell lines were previously established to study the bat immune response against viral infection. However, the lack of professional immune cells such as dendritic cells (DC) and macrophages has greatly limited the significance of current investigations. Using *Pteropus alecto (P. alecto*) GM-CSF plus IL4, FLT3L and CSF-1, we successfully generated bat bone marrow-derived DC and macrophages. Cells with the phenotype, morphology and functional features of monocyte-derived DC, *bona fide* DC or macrophages were obtained in GM-CSF/IL4, FLT3L or CSF-1 cultures, respectively. The successful generation of the first bat bone marrow-derived immune cells paves the way to unlocking the immune mechanisms that confer host resilience to pathogens in bats.

Bats serve as reservoir hosts for viruses that are related to many deadly emerging diseases in humans including Nipah virus, Hendra virus, SARS-like Coronavirus and Ebola virus^1^,^2^,^3^,^4^,^5^. Interestingly, bats carrying these viruses, which are pathogenic in humans and other mammals, show no clinical signs of diseases under natural or experimental infection conditions^3^,^6^,^7^,^8^,^9^. This unique ability may reflect an unknown interaction between these bat viruses and the bat immune system as a result of extensive co-evolution over a long period of time^10^.

Development of bat cell lines is essential for studying the bat immune system, particularly viral-host interaction under *in vitro* conditions. To this end, various non-immune bat cell lines, originating from either insectivorous or fruit-bats and covering multiple species, had been constructed including *Pteropus, Eidolon, Hypsignathus, Rousettus, Epomops, Myotis* and *Tadarida* bats^11^,^12^,^13^,^14^. These cell lines, either primary or immortalized, supported bat viral infection studies and basic host responses. In contrast to the rapid clearance or reduction of bat viruses evident in *in vivo* experiments, viral replication does not appear to be significantly different to other hosts. However, when comparing the multiple *in vitro* studies in these bat cell lines^14^,^15^,^16^, bat viruses exhibited subversion of the bat immune system^11^,^16^. These observations prompted us to ask whether these bat cells serve as an appropriate model for studying the bat immune response.

Dendritic cells (DC) are professional antigen-presenting cells that initiate and regulate the pathogen-specific adaptive immune responses and are central to the development of immunologic memory and tolerance^17^,^18^,^19^, whereas macrophages are critical effector cells and regulators of inflammation and the innate immune responses^20^,^21^. Possibly equipped with all the major innate immune recognition receptors, they can secrete cytokines, interferons and pro-inflammatory factors to activate and recruit immune cells to the site of infection upon recognition of pathogens^18^,^19^,^20^,^21^,^22^,^23^. Understanding how bat DC and macrophages respond to viruses is critical for studying bat antiviral immunology. However, while several reports characterize non-hematopoietic bat cell lines^11^,^12^,^13^,^14^, there is currently no report of successful culture or isolation of bat DC or macrophages.

In this study, we characterize the first bat bone marrow-derived DC and macrophages. We utilised overexpressed *Pteropus alecto (P. alecto*) granulocyte-macrophage colony-stimulating factor (GM-CSF) and interleukin 4 (IL-4), or FMS-like tyrosine kinase 3 ligand (FLT3L) to generate bone marrow-derived DC, while colony-stimulating factor 1 (CSF-1, M-CSF) allowed the generation of bone marrow-derived macrophages by adapting previously established protocols^24^,^25^,^26^. The successful generation of these bat immune cells will fill this urgently needed technological gap and greatly facilitate our understanding of any bat-specific immune mechanisms contributing to their resistance to viral diseases.

## Results/Discussion

### Phenotypic and morphological characterization of *P. alecto* BM-derived dendritic cells and macrophages

**Figure 1:**
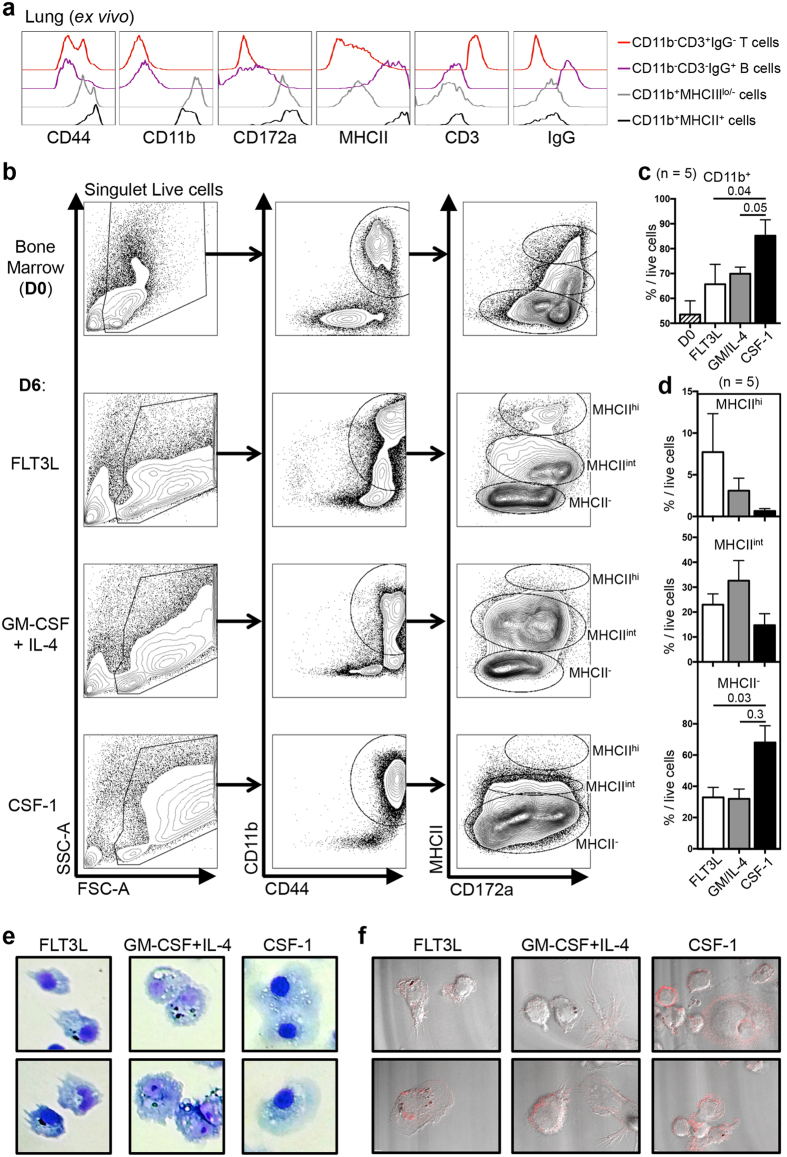
Phenotypic and morphological characterization of *P. alecto* BM-derived dendritic cells0 and macrophages. (**a**) Histogram representation of the membrane expression of CD44, CD11b, CD172a (SIRPα), MHC-II (mouse anti-IA/IE antibody), CD3 and IgG by CD11b^−^CD3^+^ IgG^+^ T cells (red line), CD11b^−^CD3^+^IgG^+^B cells (violet line), and MHC-II^−^ (grey line) or MHC-II^+^ (black line) CD11b^+^ myeloid cells (see Supplementary Fig. S1a for the gating strategy). (**b**) Flow cytometry analysis of primary *ex vivo* bone marrow (BM, Day 0, upper panels) mononuclear cells or following 6 days (D6) in culture in the presence of FLT3L, GM-CSF + IL-4 or CSF-1. Non-doublet, live cells (see Supplementary Fig. S2 for the gating strategy) were first gated based on their size (FSC-A) and granulosity (SSC-A). CD44^hi^CD11b^+^ cells were then analysed for CD172a (SIRPα) and MHC-II (anti-mouse I-A/I-E antibody) to define MHC-II^−^, MHC-II^int^ and MHC-II^hi^ cells. (**c**,**d**) Proportion of (**c**) CD11b^+^ cells among live cells or of (**d**) MHC-II^hi^ (upper panel), MHC-II^int^ (middle panel) and MHC-II^−^ (lower panel) cells, of *ex vivo* primary BM (D0, dashed filled histogram) or D6 cultured BM cells with FLT3L (white filled histogram), GM-CSF + IL-4 (grey filled histogram) or CSF-1 (black filled histogram). (**e**) Giemsa staining and (**f**) CD11b staining (red) observed by differential contrast/confocal microscopy, of D6 cultured BM cells with FLT3L (left panel), GM-CSF + IL-4 (middle panel) or CSF-1 (right panel). P values were calculated using the paired t-test.

We hypothesised that similarly to human and mouse bone marrow (BM)-derived mononuclear cells (MNC), bat BM-derived MNC would differentiate into macrophages in the presence of CSF-1, into *bona fide* dendritic cells (DC) in the presence of FLT3L, and into monocyte-derived DC in the presence of GM-CSF + IL-4^24^,^25^,^26^,^27^. Based on sequences obtained from the *P. alecto* genome^28^, we produced recombinant *P. alecto* CSF-1, GM-CSF, IL-4-GFP fusion proteins and a fusion protein comprising the functional unit of *P. alecto* FMS-like tyrosine kinase 3 ligand (FLT3L) called vaccibodies (Supplementary Fig. S1a,b). *P. alecto* FLT3L vaccibodies were initially produced to detect FLT3L-expressing cells among primary bat MNC but it showed good functional activity and, thus, was used in this study. In order to characterise BM-derived MNC by flow cytometry, we first validated that antibodies directed against human or mouse membrane molecules allowed to detect membrane molecules with a similar cellular expression pattern in *P. alecto* bat (Fig. 1a and Supplementary Fig. S1c). Antibodies previously described as cross-species reactive and targeting membrane proteins that showed good conservation between *P. alecto* human and mouse were used (Supplementary Fig. S1a). We also used an anti-CD3 intracellular domain (highly conserved across-species) and a commercial anti-bat IgG (Martìnez Gòmez *et al*., *Scientific Reports*, in press). Among live CD44^+^ lung MNC, CD11b^+^ cells could be split into MHC-II^lo/−^ and MHC-II^+^ cells while putative CD3^+^IgG^−^ T cells and CD3^−^IgG^+^ B cells could be detected among CD11b^−^ cells (Supplementary Fig. S1c). This analysis showed that both CD11b^+^MHC-II^−^ (putative monocyte/macrophages) and CD11b^+^MHC-II^+^ (putative DC) expressed high levels of another myeloid cell marker, CD172a (SIRPα), but did not express the B (IgG) and T (CD3) cell lineage markers (Fig. 1a). Using these antibodies, we next analysed *P. alecto* BM-derived MNC cultured for 6 days (D6) with *P. alecto* FLT3L vaccibodies, GM-CSF+IL-4 (GM/IL-4) or CSF-1 and compared them to the BM cells at Day 0 (D0, *ex vivo*) by flow cytometry (Fig. 1b–d, see Supplementary Fig. S1d for the total gating strategies). At D6, adherent cells from the three different culture conditions showed an increased size (FSC-A) and granulosity (SSC-A) as compared to *ex vivo* BM cells (Fig. 1b), suggesting *in vitro* acquired activation in our culture conditions. It is important to note that most cells were adherent in these three culture conditions, while this was not the case when cultured in the absence of any of these growth factors. While 53.5% of *ex vivo* BM cells (D0) expressed the myeloid marker CD11b, 65.7% and 69.9% of D6 FLT3L- and GM/IL-4- cultured cells expressed CD11b, respectively. When cells were cultured 6 days in the presence of CSF-1, the proportion of CD11b^+^ cells reached 84.8% (Fig. 1b,c). Cells were also analysed for expression of CD172a (SIRPα) and for MHC-II, with this latter molecule being expressed at an intermediate level by immature BM-derived dendritic cells (BM-DC) and at a high level by mature BM-DC obtained from both human or mouse BM^29^. From the five bats tested, among CD44^+^CD11b^+^ cells, a well-defined population of CD172a^+^MHC-II^hi^ cells was observed only when cells were cultured 6 days with the pan-DC growth factor FLT3L (Fig. 1b,d). A population of CD172a^+/lo^MHC-II^int^ cells could also be detected in both D6 FLT3L and GM/IL-4– cultured BM-derived cells, and only in low proportion in CSF-1-cultured BM-derived cells (Fig. 1b,d). Giemsa staining (Fig. 1e and Supplementary Fig. S1e) and confocal microscopy (DiC/CD11b staining, Fig. 1f) of D6 cultured cells showed that some FLT3L-treated bat BM-DC (FLT3L-DC) had the typical dendritic morphology of *bona fide* DC. Cells with such dendritic morphology were not observed in the other culture conditions (Fig. 1e, f and Supplementary Fig. S1e). GM-CSF/IL-4 cultures (GM/IL4-DC) gave rise to large, granulous cells with a rough membrane resembling monocyte-derived DC while CSF-1 cultures (CSF-1-MΦ) gave rise to cells with a classical large, foamy macrophage morphology (Fig. 1e,f and Supplementary Fig. S1e).

### T-cell allostimulatory and phagocytic capacities of *P. alecto* BM-derived DC and macrophages

**Figure 2:**
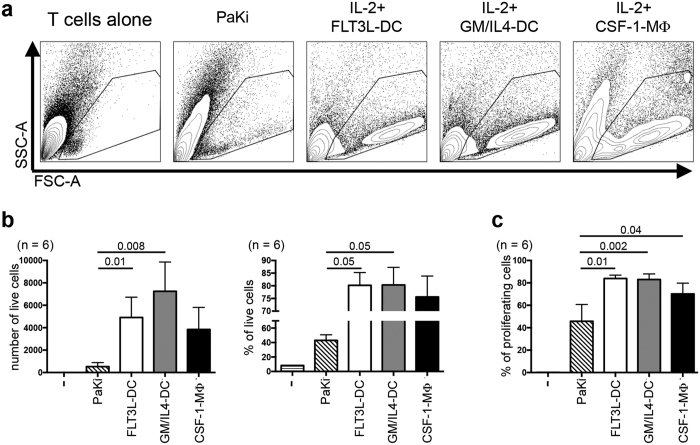
Characterization of T-cell allostimulatory capacity of *P. alecto* BM-derived DC and macrophages. (**a–c**) Following 6 days of culture of BM mononuclear cells as described in Fig. 1, a mixed lymphocyte reaction (MLR) was carried out by co-culturing these differentiated cells with CFSE-labelled allogenic *P. alecto* bat lung (n = 4) or spleen (n = 2) mononuclear cells in the presence of recombinant human IL-2 (50 UI/ml) for another 6 days until Day 12 (D12). As controls, the lung or spleen cells were cultured 6 days alone (far left panel) or in the presence of PaKiT03 cells (PaKi, left panel). At D12 (6 days of MLR), cells were analysed by flow cytometry (see Supplementary Fig. 2a for the gating strategy) and (**a**) their size (FSC-A) and granulosity (SSC-A) were analysed. (**b**) The number (left panel) and proportion (right panel) of live cells in each condition described in panel (**a**) are displayed. (**c**) Proportion of proliferating cells (CFSE^lo^, see Supplementary Fig 2 for CFSE histograms) among live cells in each condition described in panel (**a**) are displayed. P values were calculated using the paired t-test.

We next evaluated functional capacities of D6 cultured *P. alecto* BM-derived cells. A major functional specialization of DC is their strong T-cell allostimulatory capacity. To date, the only antibody capable of staining for bat T cells is the intracellular anti-CD3 (Fig. 1a), thus preventing us from purifying live bat T cells. Therefore, D6-cultured BM cells (10,000 cells) were co-cultured for another 6 days with 100,000 CFSE-labelled total allogenic lung (n = 4) or spleen (n = 2) MNC (containing T cells, see Fig. 1a; Fig. 2a–c, see Supplementary Fig. S2a for total gating strategy). As controls, lung or spleen MNC were cultured either alone or in the presence of PaKiT03 cells (*P. alecto* kidney cell line). Large (FSC-A^hi^) “blastic” cells, a classical feature of activated T cells, could be detected only when lung MNC were co-cultured with BM-derived cells, particularly with FLT3L- and GM/IL-4- cultured BM-DC, and to a lower extent with CSF-1-cultured BM-macrophages (Fig. 2a). Such blastic cells were completely absent from the lung or spleen MNC cultured alone, and only very few blastic cells could be detected in lung MNC cultured with PaKiT03 cells (Fig. 2a). The greatest cellular viability was obtained in the presence of FLT3L-DC and GM/IL4-DC (80% of live cells among total cells) with more than 3,000 viable cells detected by flow cytometry (Fig. 2b). These non-adherent blastic cells were mostly negative for CD11b (Supplementary Fig. S2), and were more numerous and had a lower granulosity than the BM-derived cells initially placed into culture. Furthermore, blastic cells were positive for CFSE (Fig. 2c and Supplementary Fig. S2a), which indicates that they were mostly derived from the allogenic lung MNC and that they did not correspond to the BM-derived DC or macrophages. While only a small proportion of lung MNC proliferated (CFSE^lo^) when cultured alone (8.3%), a strong proliferation was observed when BM-derived cells were present, particularly in the presence of FLT3L-DC and GM-CSF/IL4 cultures (84.0% and 83.05% of proliferation, respectively), and to a lesser extent in the presence of CSF-1-MΦ (70.2%) (Fig. 2c and Supplementary Fig. S2). While both the viability and number of cells recovered when cultures were done in the presence of PaKIT03 cells were low (Fig. 2a), these later were able to induce some proliferation (45.8%), but to a much lower level as compared to BM-derived DC and macrophages (Fig. 2c). Thus, this stronger allostimulatory capacity of FLT3L-DC is in line with the presence of MHC-II^hi^ (putative “mature”) cells in the BM-derived cells (see Fig. 1a,c), while GM/IL4-DC, which comprises a majority of MHC-II^int^ (putative “immature”) cells, showed a slightly lower allostimulatory capacity.

**Figure 3:**
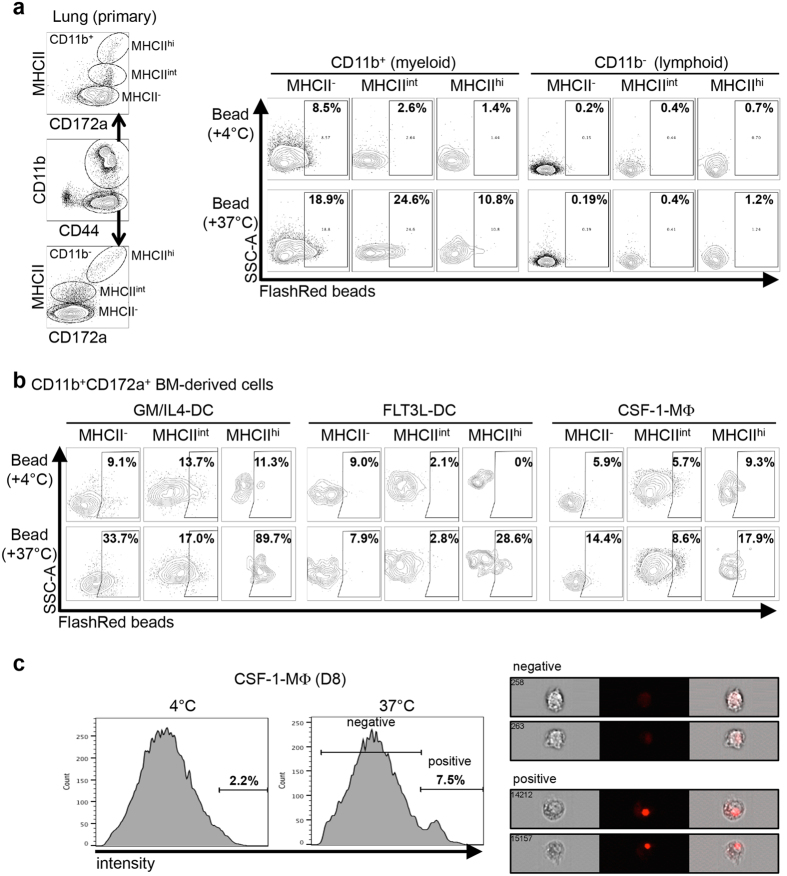
Characterization of the phagocytic capacity of *P. alecto* primary and BM-derived DC and macrophages. (**a,b**) Following 90 minutes co-culture with fluorescent FlashRed dye-conjugated polystyrene beads at +4 °C or at 37 °C, (**a**) primary (*ex vivo*) lung MNC and (**b**) 6 days-cultured BM-derived cells were analysed by flow cytometry to evaluate their phagocytic capacity. (**a**) Primary lung cells were gated first based on their expression of CD11b into CD11b^−^ lymphoid cells and CD11b^+^ myeloid cells, these two cell subsets being next gated based on MHC-II expression level into MHC-II^−^, MHC-II^int^ and MHC-II^hi^ (left panel), and analysed for their content in FlashRed beads. (**b**) 6 days cultured BM-derived cells were analysed as in Fig. 1b and their content in FlashRed beads was analysed. (**c**) Macrophages (CSF-1-MΦ) obtained from BM primary cells cultured in the presence of CSF-1 for 8 days (D8) were analysed for their phagocytic capacity of fluorescent FlashRed dye-conjugated polystyrene beads and analysed using the Amnis Imagestream. Histograms displaying the FlashRed intensity of macrophages cultured with beads at 4 °C (left histogram) or at 37 °C (right histogram) are displayed. Within histograms, the proportion of positive cells (gated as “positive”) is shown. Images of representative cells falling in the “negative” (upper right images) or in the “positive” (lower right images) gates of macrophages cultured at 37 °C are shown.

We next evaluated the phagocytic capacity of primary lung (*ex vivo*) and BM-derived MNC (Fig. 3). We first confirmed that primary lung CD11b^+^ MNC were phagocytes since they were capable of phagocytising fluorescent polystyrene beads while CD11b^−^ (lymphoid) cells were incapable to do so (Fig. 3a, see Supplementary Fig. S3a for the total gating strategy and Supplementary Fig. S3b for the no-bead control). Among CD11b^+^CD172a^+^ cells, this function was maximal in MHC-II^int^ putative macrophages, followed by MHC-II^−^ putative monocytes and finally, by MHC-II^hi^ putative cDC (Fig. 3a). Similarly to primary CD11b^+^ Lung MNC, BM-derived DC and macrophages were also phagocytic (Fig. 3b). Contrary to primary lung MNC, the greatest phagocytosis was observed in MHC-II^hi^ cells. As a control, PaKiT03 cells were analysed in parallel and did not show any phagocytic activity (Supplementary Fig. S3c). To confirm that the BM-derived cells did engulf the beads, CSF-1-MΦ cultured in the presence of fluorescent polystyrene beads were analysed using the Anmis Imagestream platform. More than 10,000 cells were analysed for each condition. When CSF-1-MΦ were cultured with beads (0.5–1 μm) at 4 °C, very few cells (2.2%) engulfed at least one bead, while 7.5% did so when culture was carried out at 37 °C (Fig. 3c). As a control, PaKiT03 cells were cultured the same way and minimal cells (1.1%) could uptake a fluorescent bead (Supplementary Fig. S3d).

### Cytokine and interferon responses of *P. alecto* BM-derived DC and macrophages

**Figure 4:**
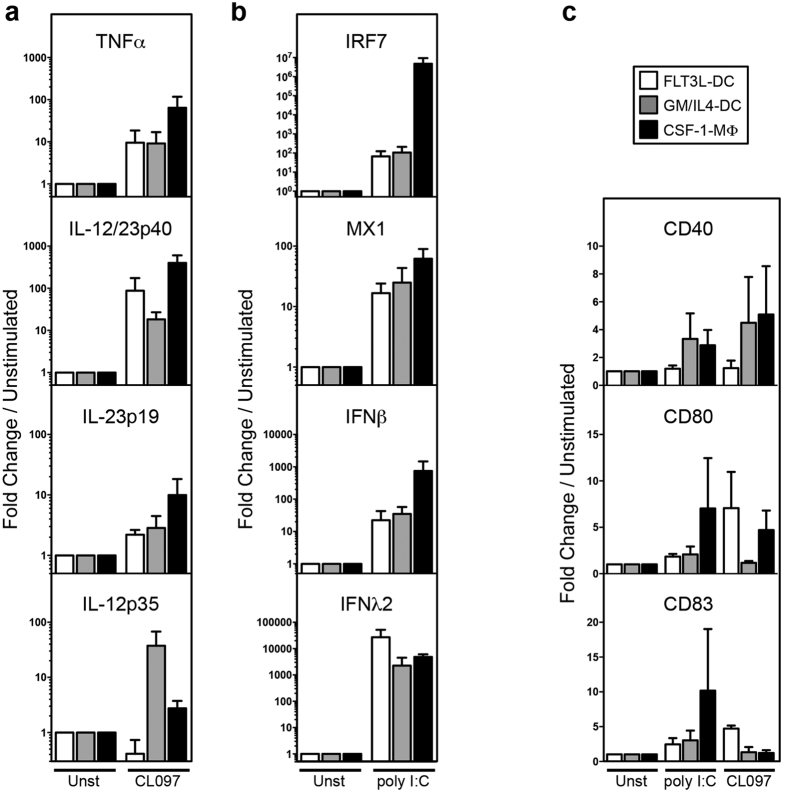
Pro-inflammatory cytokines, type-I and type-III interferon responses and costimulatory molecules induction of *P. alecto* BM-derived DC and macrophages. 8-day differentiated BM derived FLT3L-DC, GM/IL4-DC or CSF-1-MΦ cells obtained from three different *P. alecto* bats (n = 3) were left untreated or stimulated with poly I:C or CL097 for six hours. The expression level of mRNA coding (**a**) pro-inflammatory cytokines (TNFα, IL-23p19, IL-12p35 and IL-12p40), (**b**) interferon pathway molecules (IFNβ, IFNλ2, IRF7 and Mx1) and (**c**) co-stimulatory molecules (CD40, CD80, CD83) was determined by qPCR. Data were analysed using the Pfaffl method relative to the SNRPD3 housekeeping gene, then shown as fold induction above control. Data represents the average of three individual bats and error bars indicate standard error of the mean (SEM).

A major effector function of DC and macrophages is to secrete pro-inflammatory cytokines, anti-viral type I and type III interferons (IFN) as well as upregulating costimulatory molecules upon activation^23^. We examined the expression of mRNA coding for cytokines and costimulatory molecules in bat BM-derived DC and macrophages upon stimulation with Toll-like receptor 3 (TLR3) ligand (poly I:C) and TLR7/8 ligand CL097 (Fig. 4). Tumour necrosis factor alpha (TNFα), a central orchestrator of inflammation, showed the strongest induction in macrophages following TLR7/8 triggering (Fig. 4a). We next evaluated the expression of the different chains of the heterodimeric cytokines, IL-12 and IL-23. These cytokines stimulate IFNγ production and cytotoxicity by T cells and NK cells, and also participate in the polarization of naïve T cells into Th1 or Th17 cells, respectively. Bioactive IL-12p70 and IL-23 are heterodimers sharing the IL-12/IL-23p40 chain that is associated with the specific IL-12p35 and IL-23p19 chains, respectively^30^,^31^,^32^. IL-12/IL-23p40 transcripts were strongly induced in both FLT3L-DC and macrophages by CL097 and to a lesser extent in GM/IL4-DC. IL-23p19 was mostly induced in macrophages, while IL-12p35 was only strongly induced in GM/IL-4-DC. We next evaluated the expression of genes involved in type-I and type-III anti-viral IFN production (Fig. 4b). IRF7 is a transcription factor that activates the transcription of genes coding for Type-I IFN such as IFNβ and Type-III IFN such as IFNλ2. These IFNs can then stimulate the expression of IFN-stimulated anti-viral genes, including MX Dynamin-like GTPase I (MX1). Following TLR3 stimulation, MX1 transcript levels were increased in the three types of BM-derived myeloid cells while macrophages showed the strongest induction of IRF7 and IFNβ. Interestingly, FLT3L-DC showed the greatest capacity to produce IFNλ2. Poly I:C triggers IFN-III production only by *bona fide* cross-presenting mouse CD8α^+^ and human CD141^+^ conventional DC1 (cDC1)^33^. Such strong IFNλ2 production by FLT3L-generated, bat BM-derived DC indicates that they share this functional specialisation with mouse and human cDC. This observation might also indicate that the strong IFNλ2 transcript expression previously observed in bat primary splenocytes stimulated with poly I:C might have originated from primary *bona fide* cDC^34^. Costimulatory molecules (CD40, CD80 and CD83) showed variable induction levels following TLR3- (poly I:C) or TLR7/8- (CL097) triggering (Fig. 4c). TLR3-triggering induced a low increase in CD40 mRNA expression in GM/IL4-DC and macrophages only, while increase in CD80 and CD83 mRNA expression levels were only observed in macrophages. Following TLR7/8-triggering, FLT3L-DC showed the greatest increase in both CD80 and CD83 expression, while CD40 was induced also at low levels only in GM/IL4-DC and macrophages. This further indicates variation in the degree of maturation and functionality between each generated cell subtype that may be utilised for various functional assays.

## Conclusion

Here we present the first documented methodology for the production of monocyte-derived DC, *bona fide* DC and macrophages from bat bone marrow. We utilised the established method of recombinant GM-CSF/IL-4, FLT3L or CSF-1 proteins as commonly used on mouse bone marrow and applied this strategy to a wild animal species. Once these proteins are cloned from the endogenous species and expressed we believe this method could be utilised in any wild species of animal. While simplistic in nature this achievement allows investigation of viral zoonotic experiments in phenotypically functional immune cells, investigation of immune cell function and interaction between different immune cells. These resources were previously limited due to constant need of catching wild bats and the lack of research-oriented bat colonies for fresh materials. We show that such immune cells can be generated in large enough quantities for functional studies, transcriptional profiling, analysis of surface markers and even antigen presentation studies. This will facilitate further research into potential mechanisms behind bat-borne zoonotic infection “spill-over” from bats to humans or livestock. It will also open new avenues in understanding specific immune mechanisms that bats have established to reduce or eliminate clinical diseases while concurrently living with various pathogens.

## Methods

### Bats sample collection and cell isolation

All animal processing work was conducted in accordance with approved guidelines, methods and permits from the Queensland Animal Science Precinct and the CSIRO Australian Animal Health Laboratory. All experimental protocols were approved by the Duke-NUS Medical School. Capture and processing of adult bats (*Pteropus alecto*) was conducted as described previously^12^. To harvest bone marrow (BM) cells, ribs from the bats were collected and muscle tissues were stripped off the bones before rinsing them in ethanol briefly and then in RPMI 1640 (Gibco) with 10% (v/v) fetal bovine serum FBS. Caps at both ends of the ribs were then snipped off and RPMI media was used to flush out the BM through the ribs using a needle and syringe. Harvested BM cells were subsequently washed with PBS, re-suspended in RPMI 1640 with 10% (v/v) (FBS) and 7% (v/v) DMSO, and stored in liquid nitrogen. Bat lung cells were prepared as described previously^35^.

### Generation of recombinant *P. alecto* GM-CSF, IL-4 and CSF-1

*P. alecto* CSF2 (coding for CSF-2 also called GM-CSF), IL-4 and (coding for CSF-1 also called M-CSF) genes have been annotated previously^28^. The sequences were sent for gBlock synthesis (IDT) and then cloned into the pcDNA 6.2/EmGFP TOPO vector (Thermo Fisher Scientific) for expression in bat cells. Primers used to subclone *P. alecto* CSF1 and CSF2 genes from the gene block are shown in Supplementary Table S1. Of note, the CSF1 gene was human codon optimised during synthesis. Endotoxin-free maxipreps of plasmids were prepared using kits (Omega) and transfected into 5 × 10^6^ PakiT03 cells^12^ using Lipofectamine 3000 (Thermo Fisher Scientific; 10 μg/10 cm dish) according to the manufacturer’s standard protocol. Six hours later the media containing transfection reagent was replaced. After 48 h, media containing cytokines was collected and filtered using a 0.8 μm filter before storing at −80 °C until required.

### Generation of *P. alecto* FLT3L vaccibodies

Construction of the vaccibody gene construct has been previously described^36^. *P. alecto* FLT3L (acc.nr. ELK18536 aa 22–276) was synthesised by Genscript and cloned into the vaccibody gene construct using BsmI and BsiWI. mWasabi was cloned into the construct using SfiI, as previously described^36^. To express the vaccibodies, the gene constructs were transiently transfected into HEK293T cells grown in 5-layer tissue culture flasks (Falcon Multi-Flask) using Lipofectamine 2000 (Invitrogen). Vaccibodies were purified by harvesting supernatants and applied them onto a Sepharose 4 Fast Flow column (GE Healthcare) conjugated with an antibody recognizing the CH3 domain of the vaccibody (clone HP6017), as previously described^37^. Bound vaccibodies were eluted with 0.1 M Glycin-HCI pH 2.7, dialysed twice in PBS, concentrated using a 50-Kd cutoff Vivaspin column (Sartorius Stedim Biotech), aliquoted, and stored at −80 °C until use.

### 6 day-cultures of BM primary mononuclear cells to generate dendritic cells and macrophages

Frozen *P. alecto* bone marrow cells were thawed in 37 °C water bath for 30 s. Each vial of cells (1 ml) were immediately diluted with 9 ml of fresh RPMI 1640 containing 10% (v/v) FBS and spun down at 1300 rpm for 5 min. Cells were resuspended in 10 ml fresh RPMI 1640 containing 10% (v/v) FBS and incubated for 1 h at 37 °C. Three million cells were cultured in 3 ml RPMI 1640 with 10% (v/v) FBS in each well of 6-well plates. Cells were either supplied with 600 ng/ml FLT3L, 10% GM-CSF + 10% IL-4 supernatant (v/v) or 10% CSF-1 supernatant (v/v) to generate FLT3L-DC, GM/IL-4-DC or CSF-1-MΦ, respectively. At day 3 (D3), half of the media was replaced with fresh media containing 20% GM-CSF + 20% IL-4 (v/v), 1200 ng/ml FLT3L or 20% CSF-1 (v/v). At day 6 (D6), adherent cells were dislodged using ice-cold PBS containing 2 mM EDTA at room temperature. Cells were then spun down and resuspended for either flow cytometry staining, confocal staining, phagocytosis assays, mixed lymphocyte reaction or cultured at cell density of 1–2 × 10^5^ cells/well in 24-well plates for qPCR assays.

### Flow cytometry staining

The following antibodies (Ab) were used for FACS analyses: goat polyclonal anti-bat IgG (Novus Biologicals, NB7237) detected using an anti-goat IgG secondary antibody (TermoFisher), mouse IgG1 anti-human CD3-FITC (clone CD3-12, Abcam), chicken IgY anti-CADM1 (Necl2, SynCAM1, clone 3E1, MBL) detected using a donkey F(ab)’2 anti-chicken IgY-AlexaFluor647 (Jackson Immunoresearch), rat IgG2b anti-CD44-APC/eFluor780 (clone IM7, eBioscience), rat IgG2b anti-CD11b-Brilliant Violet711 (ITGAM, clone M1/70, BD Biosciences), mouse IgG1 anti-CD172a (SIRPα, clone DH59B, KingFischer Biotech Inc) detected using a polyclonal goat Ig anti-mouse IgG1-PE/Cy7 (batch Poly4053, Biolegend), and rat IgG2a anti-mouse MHCII-FITC (I-A/I-E, clone 2G9, BD Biosciences). Cells (1–5.10^6^ cells/tube) were washed and incubated with Live/Dead blue dye (30 min, 4 °C; Invitrogen/Life Technologies) in PBS. Then, 5% heat-inactivated FBS was added (15 min, 4 °C; Sigma Aldrich). Cells were labelled with antibodies, then washed and stained with secondary reagents. For CD3 intracellular staining, following secondary reagents staining, cells were fixed, permeabilized and stained with the anti-CD3 antibody using the BD Cytofix/Cytoperm kit (BD Biosciences) following manufacturer’s instructions. The samples were acquired using a FACS FORTESSA (BD Biosciences). Analyses were carried out using FlowJo V10 (Tree Star Inc).

### Cytospin and Giemsa staining

Cytospins were prepared from 6-day cultured BM-derived cells stained with Hema 3 system according to manufacturer’s protocol (Fisher Diagnostics). Cells were then stained as described previously^35^.

### Confocal microscopy

*P. alecto* bone marrow cells were seeded in 6-well plates with 1.5 glass coverslips inside during differentiation. At day 6, coverslips with cells attached were moved to 24-well plate where cells were fixed in 4% paraformaldehyde/0.37% Gluteraldehyde in TBS at room temperature for 40 minutes. After removal of fixative, cells were washed three times with TBS, followed by blocking with 5% BSA in TBS for 30 minutes. Cells were stained with anti-CD11b-Brilliant Violet711 (mentioned above, 1:200 in TBS) and Phalloidin 647 (Molecular Probes, Thermo Scientific) for 1 h. Coverslips were mounted in mowiol 4.88 in the dark overnight at room temperature. Single plane images with differential contrast (DiC) were captured on a Leica SP8 (STED3x) machine with 405/white light lasers.

### Mixed lymphocyte reaction

Allogenic *P*. a*lecto* lung mononuclear cells (MNC) were labelled with 0.2 μM carboxyfluorescein succinimidyl ester (CFSE) (Thermo Scientific) for 10 min at 37 °C. 10,000 6 day-culture BM-derived DC or macrophages were co-cultured with 100,000 CFSE labelled lung MNC in the presence of 75 UI/ml of recombinant human IL-2 (R&D Systems) for 6 days in AIM-V medium (Thermo Scientific) supplemented with 10% human AB-serum (Sigma Aldrich). As controls, lung MNC were cultured 6 days alone or in the presence of 75 UI/ml of recombinant human IL-2. On day 6, cells were analysed by flow cytometry.

### Phagocytosis assay

Approximately 1–2 × 10^5^ macrophage cells/tube (6-day culture BM with CSF-1) were placed in 500 μl of RPMI 1640 medium with 2% FBS and pre-chilled in an ice:water slurry for 5–10 minutes. Fluorescent microspheres beads (0.50–0.99 μm) (Bang Laboratories) were added at one bead per cell to the suspension in a microfuge tube and incubated either on ice or at 37 °C for 1 h. Cells were then centrifuged briefly (1,300 rpm, 4 °C for 5 min) and washed twice in 1 ml cold PBS before being resuspended in 25 μl of FACS buffer (PBS, 2% FBS, 2 mM EDTA) and analysed on the Amnis ImageStream®X Mk II imaging flow cytometer. Samples were acquired at x60 magnification and the percentage of positive/negative cells were quantified with the inbuilt IDEAS software.

### Stimulations and Quantitative Real Time PCR (qPCR)

Differentiated BM-derived DC or macrophages were dislodged using cold PBS + 2 mM EDTA at day 7. Cells were placed in a 24-well plate at 1–2 × 10^5^ cells/well and cultured with cytokines-free RPMI medium containing 10% FBS. Cells were then treated with poly I:C (Invivogen) or CL097 (Invivogen) at a final concentration of 1 μg/ml for 6 h before collection in RLT buffer (QIAGEN) for further RNA extraction. RNA was purified using the RNeasy^®^ Mini Kit (QIAGEN) with on-column DNaseI digestion step using the RNase-Free DNase Set (QIAGEN). cDNA was subsequently synthesized using QuantiTect^®^ Reverse Transcription Kit (QIAGEN). qPCR was performed in triplicates to determine transcription levels of various genes coding for TNFα, IL23-p19, IL12-p35, IL12-p40, IFNβ, IFNλ2, IRF7 and Mx1. Reactions were setup using the SensiFAST™ SYBR No-ROX Kit (Bioline) and assays were run on the CFX96 Touch™ Real-Time PCR Detection System (Bio-Rad) under the following cycling condition: 95 °C for 5 min, followed by 40 cycles of 95 °C for 5 s and 57 °C for 30 s, and ending with a melt profile analysis. Relative expression of the targeted gene was determined by REST formula, relative to the housekeeping gene SNRDP3^38^,^39^. qPCR primers for IFNβ, IFNλ2, MX1 and IRF7 have been mentioned previously^34^,^40^. All primers used in this study are listed in Table S1.

### Statistical analysis

Paired t-test was used. Differences were defined as statistically significant when p < 0.05. All these tests were performed using GraphPad Prism 6.

## Additional Information

**How to cite this article**: Zhou, P. *et al*. Unlocking bat immunology: establishment of *Pteropus alecto* bone marrow-derived dendritic cells and macrophages. *Sci. Rep.* **6**, 38597; doi: 10.1038/srep38597 (2016).

**Publisher's note:** Springer Nature remains neutral with regard to jurisdictional claims in published maps and institutional affiliations.

## Supplementary Material

Supplementary Figures
