# Supplementary material for: Unlocking bat immunology: establishment of Pteropus alecto bone marrow-derived dendritic cells and macrophages
Source: Sci Rep. 2016 Dec 9;6:38597. doi: 10.1038/srep38597 (PMC5146944; doi:10.1038/srep38597)
Supplement: Supplementary Figures [file srep38597-s1.pdf]

Unlocking bat immunology: establishment of *Pteropus alecto* bone marrow-derived dendritic cells and macrophages

Peng Zhou[1,#], Yok Teng Chionh[1,#], Sergio Erdal Irac[1], Matae Ahn[1], Justin Han Jia Ng[1], Even Fossum[2], Bjarne Bogen[2,3], Florent Ginhoux[1,4], Aaron Irving[1,§,*], Charles-Antoine Dutertre[1,4,§,*], Lin-Fa Wang[1,§]

Supplementary materials

SUPPLEMENTARY TABLE S1

Targeted Gene	Sequences	Annealing temperature (°C)	Amplicon length
CD40	Fwd 5’ CCTGTTTCCTGATCTGGCCTC 3’	57	365
	Rev 5’ TTCAAAAATGGCGCAAGGGC 3’	57	
CD80	Fwd 5’ AAAGACCAGCCAGCACCAT 3’	57	434
	Rev 5’ CTTCCTTCCTGGGTGACTGTT 3’	56	
CD83	Fwd 5’ CCACTTTTTGCGAGCAGG AC 3’	55	344
	Rev 5’ TCACAGCTTGGCCTG TCAAT 3’	50	
SNRPD3	Fwd 5’ AGGTATACATCCGTGGCAGC 3’	56.9	144
	Rev 5’ CCACTTGGGCCTTCAGAATA 3’	54.5	
IL-23p19	Fwd 5’ TGTGGATCTACCAAGAGAAG 3’	51	149
	Rev 5’ CTCGTAAAACACCAGGCCCTGGT 3’	62.9	
IL-12p35	Fwd 5’ TATGAGGACTTGAAGATGTACCAG 3’	53.7	129
	Rev 5’ CAGGGCCTGCATTAGCTCATC 3’	58.2	
IL-12p40	Fwd 5’ GGTCTTAGGCTCTGGCAAAACC 3’	58.2	129
	Rev 5’ CCATCTTCCTTTTTGTGAAGC 3’	52.2	
TNFα	Fwd 5’ GAACTCCCCAAGAAGGCAG 3’	55.6	152
	Rev 5’ CCAGTCGGGAACTCTTCTC 3’	54.6	
IL4 cloning	Fwd 5’ ATGGGTCTCACCTCCCAG 3’	56.3	408
	Rev 5’ GCTCCAACTCTTTGAGTA 3’	48.7	
GM-CSF cloning	Fwd 5’ ATGTGGCTACAGAACCTGC 3’	55	432
	Rev 5’ CTTCTGCACTTGCTCC 3’	50.4	
CSF1 cloning	Fwd 5’ ATGACGGCCAGAGGAGCAGCCGGG 3’	69.2	1659
	Rev 5’ GACGGGCAGTTCCACCTGTCTGTCCTC 3’	65.8	

SUPPLEMENTARY FIGURE LEGENDS

Figure S1.

(**a**) Percentages (%) of identity in protein sequence between predicted *P. alecto* (Ptal) and human (Hosa) or mouse(Mumu) for molecules stained in flow cytometry and confocal microscopy experiments, or for soluble mediators used to generate BM-derived cells. (**b**) Schematic structure of homodimeric *P. alecto* FLT3L-mWasabi fusion protein (Vaccibody). Targeting (*P. alecto* FLT3L), dimerization (hinge and Ch3 from Human IgG3), and functional (mWasabi fluorescent protein) units are indicated. (**c**) Gating strategy for the analysis of primary lung mononuclear cells from a wild *P. Alecto* bat. (**d**) Following two steps of doublet exclusion (SSC-W/SSC-A followed by FSC-A/FSC-H), dead cells (Live/Dead^{hi}) were excluded. Live cells were then gated based on their size (FSC-A) and granulosity (SSC-A). CD44^{hi}CD11b^{+} cells were next analysed for CD172a (SIRPα) and MHCII (anti-mouse I-A/I-E antibody) to define MHC-II^{-}, MHC-II^{int} and MHC-II^{hi} cells. (**e**) Large field images of Giemsa stainings carried out on BM-derived FLT3L- (upper panel), GM-CSF + IL-4- (middle panel) and CSF-1- (lower panel) cultured cells from which the cropped images of fig. 1d were extracted (delimited by black squares).

Figure S2.

Gating strategy for the analysis of D12 MLR. Following two steps of doublet exclusion (SSC-W/SSC-A followed by FSC-A/FSC-H), cellular debris were excluded (FSC-A/SSC-A). Dead cells (Live/Dead^{hi}) were then excluded and live CD44^{+} (CD44/SSC-A) cells were analysed for the expression of CFSE (CFSE/counts histograms). Cells included in the CFSE^{lo} gate (displayed in each histogram) were defined as proliferating.

Figure S3.

(**a**) Full gating strategy to define lung primary cell subsets displayed in Fig. 3b. (**b**) The “no bead” control to Fig. 3b (Lung primary cells) data are displayed. (**c**) PaKiT03 cells were cultured 90 minutes with fluorescent FlashRed dye-conjugated polystyrene beads on ice (+4°C) or at 37°C

and analysed by flow cytometry as in Fig. 3b,c for their capacity to phagocytose FlashRed beads. The levels of FlashRed are displayed as contour plots (FlashRed beads / SSC-A; percentage of positivity displayed in each contour plot). (**d**) Cells (PaKiT03 cell line) derived from *P. alecto* kidney were analysed for their phagocytic capacity of fluorescent FlashRed dye-conjugated polystyrene beads and analysed using the Amnis Imagestream, in the same experiment as CSF-1-MΦ of Fig. 2d. Histograms displaying the FlashRed intensity of PaKiT03 cells cultured with beads at 4°C (left histogram) or at 37°C (right histogram) are displayed. Within histograms, the proportion of positive cells (gated as “positive”) is shown. Images of representative cells falling in the “negative” (upper right images) or in the “positive” (lower right images) gates of PaKiT03 cells cultured at 37°C are shown.

a

Cell marker	% identity Ptal / Hosa	% identity Ptal / Mumu
CD3	66	65
CD172a	76	66
CD11b	78	75
MHC-II	70	58
CD44	70	64
FLT3	86	81
M-CSF (CSF1)	72	64
GM-CSF (CSF2)	58	75
IL4	68	42

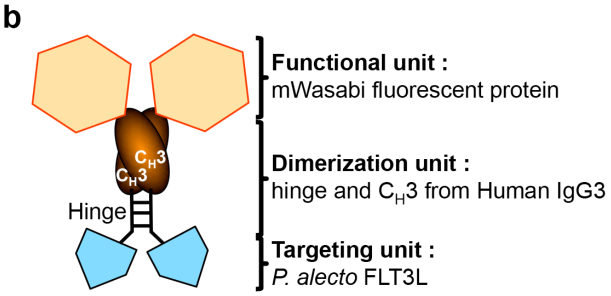

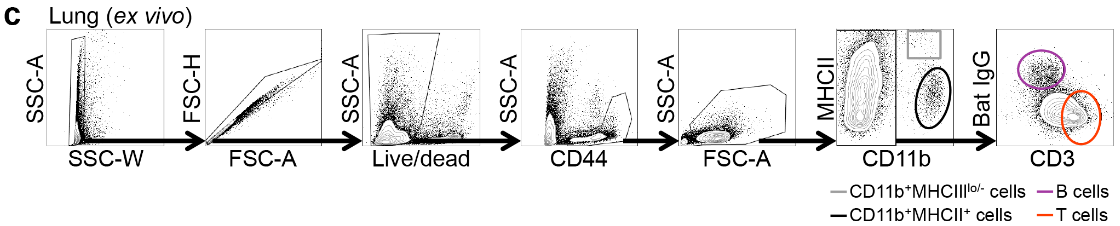

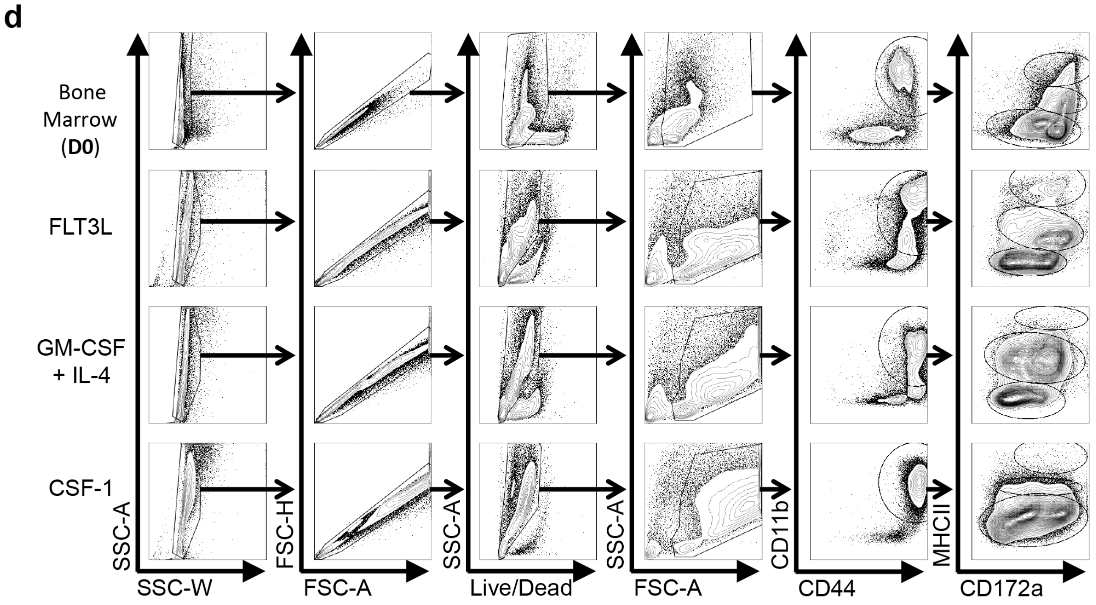

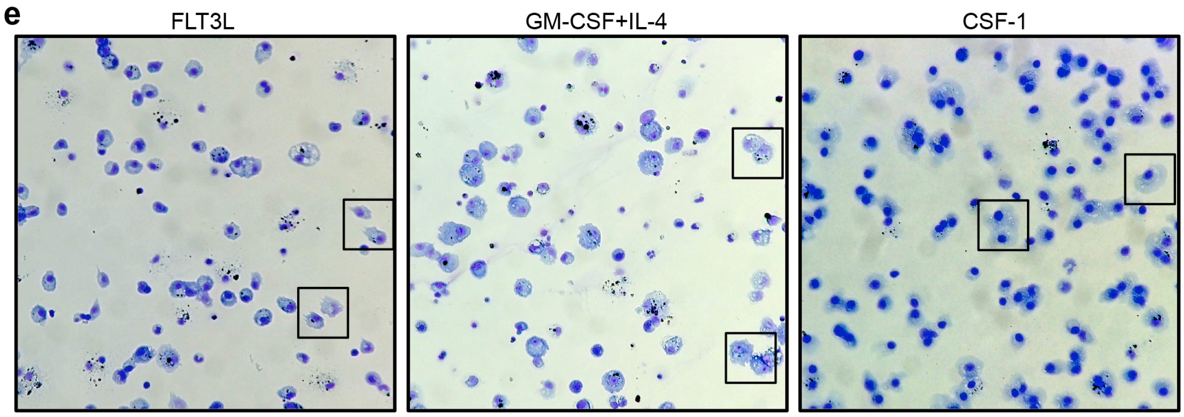

Fig. S1

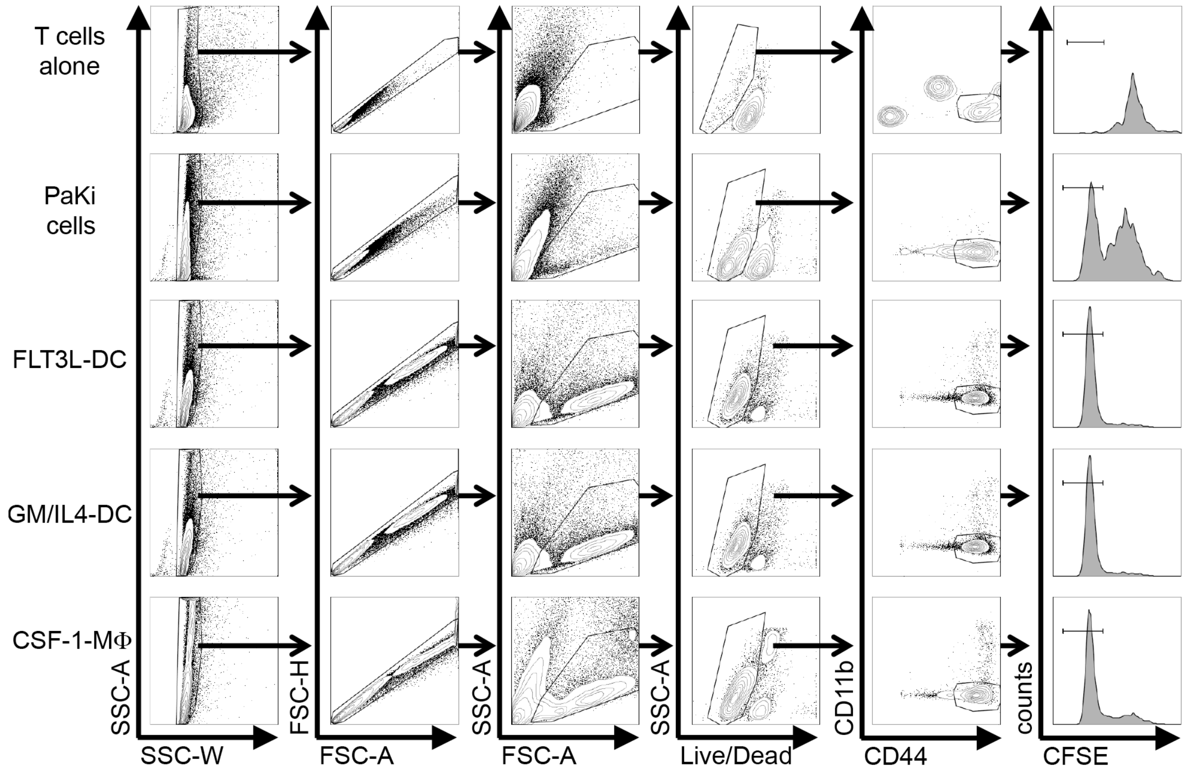
T cells alone
PaKi cells
FLT3L-DC
GM/IL4-DC
CSF-1-MΦ
SSC-A
SSC-W
FSC-H
FSC-A
SSC-A
FSC-A
SSC-A
Live/Dead
CD11b
CD44
counts
CFSE

Fig. S2

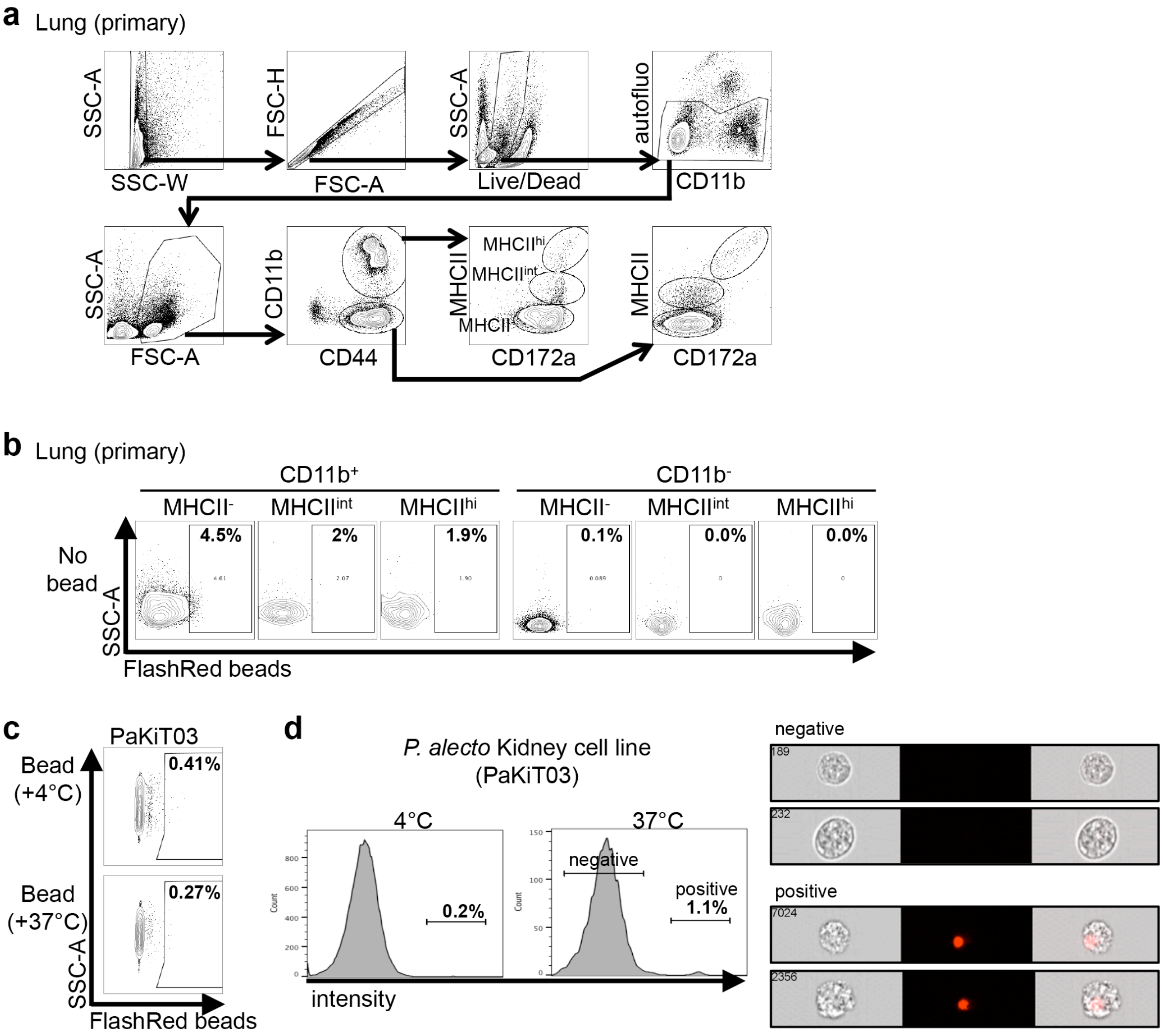
a
Lung (primary)
SSC-A
SSC-W
FSC-H
FSC-A
SSC-A
Live/Dead
autofluo
CD11b
SSC-A
FSC-A
CD11b
CD44
MHCII
MHCIIhi
MHCIIint
MHCII-
CD172a
MHCII
CD172a
b
Lung (primary)
CD11b+
CD11b-
MHCII-
MHCIIint
MHCIIhi
MHCII-
MHCIIint
MHCIIhi
No bead
4.5%
2%
1.9%
0.1%
0.0%
0.0%
SSC-A
FlashRed beads
c
PaKiT03
Bead (+4°C)
0.41%
Bead (+37°C)
0.27%
SSC-A
FlashRed beads
d
P. alecto Kidney cell line (PaKiT03)
4°C
37°C
0.2%
negative
positive
1.1%
intensity
negative
positive

Fig. S3
